# Knowledge of Nonmedical Individuals about Cardiopulmonary Resuscitation in Case of Cardiac Arrest: A Cross-Sectional Study in the Population of Jeddah, Saudi Arabia

**DOI:** 10.1155/2019/3686202

**Published:** 2019-01-16

**Authors:** Fadi Jandali Qara, Loui K. Alsulimani, Maged M. Fakeeh, Diyaa H. Bokhary

**Affiliations:** King Abdulaziz University, Jeddah, Saudi Arabia

## Abstract

**Introduction:**

In cardiac arrest victims, providing a high-quality cardiopulmonary resuscitation (CPR) is a fundamental component of initial care, especially in the out-of-hospital settings. In this study, we sought to assess the knowledge of nonmedical people regarding cardiopulmonary resuscitation in the case of out-of-hospital cardiac arrest.

**Methods:**

A cross-sectional survey containing 22 questions was administered to individuals aged ≥ 18 years, who were not health care providers. Sample included residents of Jeddah, Saudi Arabia. The survey included knowledge about cardiac arrest findings, previous experience with CPR, knowledge of basic life support (BLS), and concerns related to CPR.

**Results:**

The fully completed survey forms of 600 respondents were analysed. Out of these, 28.7% stated that they had previously received training in CPR. Regarding manifestations of cardiac arrest, 40.7% suggested loss of consciousness, 36.8% suggested cessation of breathing, and 24.7% suggested cessation of circulation. Only 11.7% among respondents were found to be able to perform chest compressions. Also, only 9.2% could perform mouth-to-mouth ventilation, and 29.5% were able to perform both. While 55.5% knew the location for performing chest compressions, 44.7% knew the correct depth, and only 18.5% knew the correct compression–ventilation rate. Bystander CPR had been performed by only 10.7%.

**Conclusion:**

In our sample, we found lack of knowledge regarding CPR. We advise for a coordinated national effort to improve the public awareness about CPR performance. This may include mass education, specialized training, and setting legislations.

## 1. Introduction

Sudden cardiac arrest is the main cause of death for millions of people worldwide every year [1, 2]. In cardiac arrest victims, cardiopulmonary resuscitation (CPR) is a fundamental component of initial care. The quality of delivered CPR improves the survival rate from cardiac arrest [3], especially when the incident happens out of the hospital.

The incidence of adult out-of-hospital cardiac arrest (OHCA) is estimated to be 95.9 cases per 100,000 persons per year [4, 5]. In a survey performed in a busy street in a city of a western region of Turkey, 40.7% of people living in a highly educated region reported having received CPR training and 3.6% had previously performed bystander CPR [2]. This requires an improvement of community awareness about recognizing the signs of cardiac arrest and determining when to start performing CPR.

There is a significant impact of having good public awareness about how to practice CPR. In the majority of cases, the first person to act in OHCA is a victim's relative, usually a nonmedical person. Approximately 70% to 75% of cases of OHCA are witnessed by nonmedical people [6–8]. The early recognition of OHCA, early activation of emergency medical services (EMS), and early provision of bystander basic life support (BLS) are the most important factors that determine the survival probability in patients with OHCA; these actions depend entirely on the knowledge, attitude, and actions of the bystanders [7, 9]. Survival after OHCA is low (3%–30%) but showed improvement after increased public education and increased use of automatic external defibrillators [7, 10]. Data collected on survival rates showed that OHCA patients who receive bystander CPR have a greater chance of survival versus those who do not [4].

Unfortunately, there are scarce data available about OHCA and CPR performed by individuals who are not health care providers in Saudi Arabia [11]. One study looked at cases of OHCA and identified differences in clinical variables and survival rates in patients arresting out-of-hospital and in the emergency department at a single hospital in Riyadh, Saudi Arabia. The study found that a total of 61 adults arrested out of hospital, and the most prevalent reported presumed cause of arrest was of cardiac origin in 82.3% of patients [11]. Patients arrested at home composed 74% of the study population with an overall mortality rate of 95.8% and a low rate of bystander CPR being performed. A family member most often transported the patient to the hospital [11].

We believe that the knowledge of nonmedical people (i.e., individuals who are not health care providers) to recognize and intervene by doing CPR in the kingdom of Saudi Arabia has not previously been evaluated. In the present study, we aimed to assess the knowledge of nonmedical people in Jeddah to recognize and intervene by doing CPR in the case of OHCA.

## 2. Methods

This cross-sectional survey study was initiated after receiving ethics committee approval from the Unit of Biomedical Ethics at King Abdulaziz University (reference no. 181-18).

Our sample size was 600 persons, which was calculated by the use of OpenEpi (Massachusetts Institute of Technology, Cambridge, MA, USA). All of the selected participants live in Jeddah, the second largest city after Riyadh and the most cosmopolitan city in the kingdom of Saudi Arabia. Prior to filling out the questionnaire, the purpose of the study was explained and verbal consent was obtained from all of the participants in the study. The inclusion criteria were an age of older than or equal to 18 years and not being a health care provider. Those who were younger than 18 years, health care providers, or who refused to participate were excluded.

The questionnaire was distributed to residents of Jeddah, Saudi Arabia, between March 2018 and April 2018. The questionnaire survey used in the present study was taken from an investigation done in Izmir, Turkey [2], and translated into Arabic by an expert in translation and subsequently translated into English by another expert in translation. Both translated versions were reviewed for consistency and clarity. Both the Arabic and the English questionnaires were distributed at separate times to 30 participants who spoke both English and Arabic fluently. The results were the same on both occasions.

The questionnaire included 22 questions in the Arabic language (which were adapted according to our practices and culture) on the demographic features of the participants; their knowledge regarding cardiac arrest manifestations (e.g., cyanosis, difficulty in breathing, nausea, and faintness of the skin); their previous CPR experiences (e.g., witnessing cardiac arrest, conducting CPR, and having a CPR certificate); whether or not they knew the national phone number for emergency situations; their BSL knowledge (e.g., compression location, number, and rate of ventilations/compressions); their concerns regarding CPR (e.g., possibility of making a mistake, contracting a contagious disease from blood and vomitus, and legal concerns); and their attitudes (i.e., who are the people to whom they will give CPR without hesitation) (**Supplementary Materials** (available here)).

Correct cardiac arrest findings and algorithms of BLS were determined according to the 2015 European Resuscitation Council Resuscitation Guidelines [12], the 2017 American Heart Association Focused Update on Adult BLS and CPR Quality [13], and the 2010 Saudi Heart Association Guidelines on CPR.

The data were analysed using the Statistical Package for the Social Sciences version 15.0 software package program (IBM Corp., Armonk, NY, USA). Categorical data were displayed as numbers (n) and percentages (%), while numerical data were displayed in the form of mean ± standard deviation (after testing for normality of distribution). The chi-squared test was used in the analysis of categorical data. A p value of < 0.05 in the statistical tests was accepted as being significant.

## 3. Results

### 3.1. Demographic Data

Out of 600 participants, 360 (60%) were females. The mean age of our population was 37.4 years ± 13.6 years. Two hundred forty-nine (41.8%) people were employees. The number of participants who stated that they had received CPR training was 172 (28.7%). Additional collected demographic data are presented in Table 1.

### 3.2. Regarding the Prodromal Symptoms and Signs of Sudden Cardiac Arrest

The main reported manifestation was chest pain (68.8%), followed by difficulty in breathing (50.8%), loss of consciousness (40.7%), and discontinuation of breathing (36.8%). The rest of the manifestations suggested by the respondents to indicate sudden cardiac arrest are presented in Table 2.

### 3.3. Identification of Cardiac Arrest Findings

With respect to consciousness evaluation, the percentage of those who knew one of the procedures correctly was 44.8%, followed by those knowing two of the procedures correctly being 25.2%, and then those knowing three of the procedures correctly being 12.8%. Regarding respiration evaluation, the percentage of those knowing one of the procedures correctly was 39.2%, followed by those knowing two of the procedures correctly (31.2%), and then those knowing three of the procedures correctly (20.2%). Furthermore, for circulation evaluation, the percentage of those knowing one of the procedures correctly was 51.4%, followed by those knowing both of the procedures correctly (32.2%). More than half of the respondents (57.2%) knew the correct meaning of chest compression, that is, ‘to apply strong compressions to the chest at certain intervals (compression)'. These results are presented in Table 3.

### 3.4. Witnessing a Sudden Death

Only 95 (15.8%) reported witnessing a sudden death before, while only 36 (21.1%) were able to perform CPR. The rest of these results are presented in Table 4.

### 3.5. CPR Attitudes

For the respondents who answered the question ‘to whom would you apply CPR without hesitation,' 465 (94.2%) stated for a family member, followed by 397 who said for a friend (66.2%), and then 313 who said for a neighbour (52.2%). For the question ‘what would you do in the case of a family member,' ‘call an ambulance' was selected by 44.4% and ‘begin to give a chest compression' was selected by 35.8%. For the question ‘what would you do regarding a stranger,' ‘call an ambulance' was reported by 54.7% and ‘begin to give a cardiac chest compression' was stated by 24.5%. The main reason that may prevent someone from giving a chest compression to either a friend/relative or a stranger was reported as ‘making a mistake' by 71.0% and 62.0%, respectively. The rest of these results are presented in Table 5.

### 3.6. CPR Knowledge and Training Status

Only 196 (32.7%) of the participants reported knowing how to give a chest compression and only 172 (28.7%) had received formal training. Regarding where these individuals were trained, 46 (21.3%) reported receiving training ‘at a course given by the trainers of the ministry of health.' The rest of these results are presented in Table 6.

### 3.7. CPR Skills

Less than one-third (177; 29.5%) reported that they could ‘both ventilate and give a chest compression.' Only 69 (11.5%) knew the proper rate of chest compression to artificial ventilation during CPR (30/2 compression/breath) and only 71 (11.8%) knew the proper rate of chest compressions (i.e., at least 100 times per minute). Of the total participant cohort, 333 (55.5%) knew the applying place of chest compression (middle of the chest) and 267 (44.5%) knew the appropriate force to apply (moderate force, such that the rib cage moves down 5 cm to 6 cm). Additionally, 428 (71.3%) knew about the defibrillator (a device that restarts a heart that has stopped working) and 132 (22.0%) knew where an automated external defibrillator or a pacemaker can be found. The rest of these results are presented in Table 7.

### 3.8. Association between Identification of Cardiac Arrest Findings and CPR Training

The survey results revealed a significant difference in the identification of cardiac arrest findings between respondents with previous CPR training and those without. Those who had received training showed a higher rate of knowing the correct procedures of evaluation of consciousness, respiration, and circulation (p = 0.151, p = 0.018, and p = 0.026, respectively). For more details, see Table 8.

### 3.9. Association of Response to a Sudden Death with CPR Training

The results revealed no significant difference in the action of the witness of a sudden death between respondents with previous CPR training and those without. For more details, see Table 9.

### 3.10. Association of CPR Application with CPR Training

The results revealed a significant association between the application of CPR and having CPR training. Those who received training showed a higher rate of knowing the correct answers regarding proper rate of chest compression /artificial ventilation during CPR, where the chest compression must be applied, the rate of chest compressions, and the force that must be applied during chest compression (p < 0.0001, p < 0.0001, p < 0.0001, and p = 0.004, respectively). For more details, see Table 10.

## 4. Discussion

It is agreed largely that CPR is one of the most essential methods available to save a cardiac patient's life. As such, we think that it is very important to evaluate the level of knowledge in our society. In the present study, we sought to assess the knowledge of nonmedical people in Jeddah to recognize and intervene by doing CPR in the case of OHCA.

At the beginning of our analysis, we calculated the percentage of nonmedical people who had participated in a CPR course in the past. Unfortunately, we found that only 172 (28.7%) individuals undertook a CPR course, despite the excellent education level that our people generally have. This finding was almost similar to those of two studies conducted in Ireland and New Zealand, respectively, which revealed percentages of 28% and 27%. However, other previous studies showed higher rates of CPR training. Specifically, 79% were reported to have CPR training by a study conducted in Washington [14], 75% in Poland [15], 69.4% in Slovenia [7], 64% in Australia [16], and 40.3% in Turkey [2]. These disparate findings can be explained by the fact that BLS courses are a mandatory requirement before getting a driver's license or before enrolment in some jobs in certain countries like Turkey and Slovenia [2, 7]. In our country, at this time, it is not a mandatory requirement, except in a very few number of workplaces. One-quarter of trained nonmedical people in the present study got their CPR experience through the Internet, television, or media, which could explain the huge gap between us and the aforementioned countries.

The methods of assessing for cardiac arrest varied between respondents who received BLS course and those who did not in a statistically significant manner. Among those who previously received CPR training, 16.9%, 28.5%, and 17.4% can properly assess consciousness, breathing, and circulation, respectively. Our findings were almost equal to those of the Slovenian study, in which the authors found that 22%, 29.4%, and 9.5% of the trained people could properly assess consciousness, breathing, and circulation, respectively [7]. On the other hand, our results were much better than those reported in a Turkish study in which the investigators found that only 5%, 5.2%, and 7.1% of the trained people could properly assess consciousness, breathing, and circulation, respectively [2]. These findings assert the good quality of the CPR training that nonmedical people in our study received.

Regarding BLS application, 29.5% can perform both breathing and cardiac compression, which was similar to the results of the Turkish study (28.7%) but less than the results of the Slovenian study (38%). In comparison, 38.8% reported that they do not know how to do either one of these actions, a finding which was similar to the Turkish study (38.6%) and lower than the Slovenian study (45.4%) [2, 7]. Additionally, when our study population was asked about the details of breathing and cardiac compression (for example, compression place, compression force, compression rate, and compression to breathing ratio), our population showed the same level of knowledge as did those participants in previous studies of other countries, which was very low in general with a clear significant difference between people who received training versus those who did not receive training. This raises the importance of the introduction of an urgent intervention to boost the level of knowledge in order to save as many cardiac arrest patients as possible.

According to our results, 15.8% of our population has witnessed a sudden death, with the majority of those patients being family members (70%). Unfortunately, only 22.1% started CPR in these cases. Similarly, in the Turkish study, 18.6% of the participants reported having witnessed a sudden death and 22% said they started CPR, with the main reason behind not conducting CPR being fear to make a mistake, which could be explained by the low level of knowledge among their population [2]. However, much lower rates of conducting CPR were reported by a study in Japan, in which 19% of participants witnessed a sudden death but only 4% of them started CPR [17]. Thankfully, a good number of people in Saudi Arabia report feeling comfortable regarding giving CPR to a member of their family (94.2%) and to a stranger (36.7%). Similarly, in a study conducted in Arizona, 80% of 750 participants reported feeling fine to perform CPR on a member of their family and 50% reported the same regarding a stranger [18].

When we put our participants in a situation of sudden cardiac arrest of one of their family members or friends, nearly one-third of participants (35.8%) would begin CPR. The most common reason for not conducting CPR was reportedly to be the fear to make a mistake (71.0%). A study conducted in Japan found that the scare of disease transmission was the most common cause (28%) [17].

On the other hand, when we asked our participants about the same situation occurring to strangers, the percentage of those willing to begin CPR decreased to 24.5%, though the most common cause remained a fear of performing it the wrong way (62%). However, the scare of disease transmission and legal issues increased to become 5.2% and 9.85%, respectively. In the study in Arizona, concerns about making a mistake was the most common cause of unwillingness to perform CPR (22.8%), while 17.7% reported hesitation due to potential legal issues [18]. In Japan, the most common cause was scare of transmission of diseases in 63% [17]. Again, these findings raise the value of finding a solution to increase the knowledge about CPR and correct existing mistaken beliefs.

## 5. Conclusion and Recommendations

The level of knowledge of our community regarding CPR is limited and very shallow compared to the knowledge levels of other neighbouring countries. Furthermore, some mistaken beliefs do exist. For that reason, we have some recommendations that we expect will increase the knowledge regarding CPR in Saudi Arabia. First of all, we highly recommend undertaking huge efforts to increase the awareness and knowledge of CPR. For example, we advise high-grade schools and universities to conduct a BLS course every year and to make it a routine requirement. We additionally advise our government to designate a BLS course as an obligatory requirement before getting a driver's license or prior to enrolment in some jobs that include contact with other people. Second, we suggest establishing a routine examination of CPR awareness and knowledge, which will assess the benefits of the conduction of BLS courses and attempt to elucidate the weak points so as to further improve the courses in the future. Finally, we believe it is also very important to assess the quality of CPR that has been performed, as this is a very important element in the success of resuscitation [5].

## Figures and Tables

**Table 1:** Demographic data.

**Variable **	**Mean ± SD**	**Range**
**Age (years)**	37.4 ± 13.6	(18–72)

**Variable**	**N**	%

**Gender**		

Male	240	40.0

Female	360	60.0

**Education**		

Elementary	5	0.8

Intermediate	26	4.4

High school	82	13.7

College	59	9.9

University	158	26.4

Postgraduate	268	44.8

**Occupation **		

Unemployed	30	5

Worker	16	2.7

Employee	249	41.8

Housewife	117	19.7

Student	104	17.5

Retired	57	9.6

**Table 2:** Prodromal symptoms and signs of sudden cardiac arrest.

**Variable **	**N**	%
Loss of consciousness	244	40.7

Discontinuation of breathing	221	36.8

Discontinuation of circulation	148	24.7

Cyanosis	185	30.8

Nausea	66	11

Difficulty in breathing	305	50.8

Chest pain	413	68.8

Faintness of the skin	137	22.8

The individual is not moving	142	23.7

**Table 3:** Identification of cardiac arrest findings.

**Variable **	**N**	%
**Consciousness evaluation**		

No response when called	373	62.2

No response when touched	221	36.8

Not moving at all	208	34.7

I do not know	114	19

Those knowing three of the procedures correctly	77	12.8

Those knowing two of the procedures correctly	151	25.2

Those knowing one of the procedures correctly	269	44.8

Those who do not know any of the procedures (I do not know)	103	17.2

**Respiration evaluation**		

Not having any respiratory movement	360	60

Not having any respiratory sound	267	44.5

No coming air out of the mouth of an individual	310	51.7

No steaming up of a mirror placed in front of the mouth of an individual	135	22.5

I do not know	42	7

Those knowing three of the procedures correctly	115	20.2

Those knowing two of the procedures correctly	188	31.2

Those knowing one of the procedures correctly	235	39.2

Those who do not know any of the procedures (I do not know)	38	6.3

**Circulation Evaluation**		

Not feeling a pulse in the vessels of the neck	121	20.2

Not feeling a pulse in the vessels of the arm	187	31.2

Both	193	32.2

I do not know	108	18

**‘Chest compression' means**		

To scrub the chest at certain intervals	67	11.2

To apply strong compression to the chest at certain intervals (compression)	343	57.2

To scrub the heart directly by opening the chest wall	11	1.8

To apply compression directly to the heart by opening the chest wall	24	4.0

I have no idea	155	25.8

**Table 4:** Witnessing a sudden death.

**Variable **	**N**	%
**Who was he/she?**		

Somebody from my family	63	10.5

Somebody from my friends or acquaintances	14	2.3

A stranger	23	3.8

I have not seen a sudden death happen	505	84.2

**What did you do in the situation?**		

I began to give chest compressions	21	15

I conducted mouth-to-mouth ventilation	10	7.1

I both gave chest compressions and conducted mouth-to-mouth ventilation (i.e., I gave CPR)	36	22.1

I called an ambulance	57	40.7

I told somebody to call for help	42	30.7

I called for help by telephone	20	14.3

I just watched and left	25	17.9

**Table 5:** Taking action.

**Variable **	**N**	%
**If sudden death occurs in the following people, for whom would you conduct respiration and give chest compression?**		

Someone from the family	565	94.2

Friend	397	66.2

Neighbour	313	52.2

A youth in the sports hall	230	38.3

A stranger in the supermarket	220	36.7

A person who has poor personal hygiene at the bus stop	172	28.7

A gamin who is drug-dependent and uses glue, hashish, and/or heroin	154	25.7

**If somebody from among your family members or friends felt faint (sudden death), what would you do?**		

I would begin to give chest compression	215	35.8

I would call an ambulance	264	44.4

I would call somebody or call for help	119	19.8

I would just watch and leave	2	0.3

**If you witness a stranger feeling faint**,** what would you do?**		

I would begin to give chest compression	147	24.5

I would call an ambulance	328	54.7

I would call somebody or call for help	120	20.0

I would just watch and leave	2	0.5

**What concerns may prevent you from giving chest compression to your friends or relatives?**		

Making a mistake	426	71.0

Causing bone fractures	27	4.5

Causing harm to organs	25	4.2

Stopping a working heart	19	3.2

Punishment due to legal reasons	26	4.3

Contamination by blood or vomiting	5	0.8

Contracting a contagious disease	12	2.0

Other	60	10.0

**What concerns may prevent you from giving chest compression to a stranger?**		

Making a mistake	372	62.0

Causing bone fractures	19	3.2

Causing harm to organs	24	4.0

Stopping a working heart	17	2.8

Punishment due to legal reasons	59	9.8

Contamination by blood or vomiting	12	2.0

Contracting a contagious disease	31	5.2

Other	66	11.0

**Table 6:** CPR training.

**Variable **	**N**	%
**Do you know how to give chest compression in the case of cardiac arrest and respiratory standstill?**		

No	404	67.3

Yes	196	32.7

**Have you received any training?**		

No	428	71.3

Yes	172	28.7

**Where (n = 172)?**		

At school	23	10.6

At university	32	14.8

During my military service	5	2.3

At a resuscitation society course	13	6.0

At a course given by the trainers of the ministry of health	46	21.3

In a sports club	6	2.8

At a course given in the workplace	37	17.1

Television/Internet/media	53	24.5

**Table 7:** Application of CPR.

**Variable **	**N**	%
**What BLS applications can you apply?**		

I can open the airway	58	9.7

I can control respiration	7	1.2

I can ventilate/conduct mouth-to-mouth ventilation	55	9.2

I can give chest compression	70	11.7

I can both ventilate and give chest compression	177	29.5

I do not know	233	38.8

**What is the proper rate of chest compression /artificial ventilation during the chest compression (compressions/breaths)?**		

5/1	173	28.8

15/2	131	21.8

30/2	69	11.5

Other	227	37.8

**Which of the following areas must chest compression be applied on?**		

An upper part of the chest	110	18.3

Middle of the chest	333	55.5

A lower part of the chest	83	13.8

Other	74	12.3

**What must be the rate of the chest compression?**		

At least 150 times per minute	22	3.7

At least 100 times per minute	71	11.8

At least 50 times per minute	162	27.0

I don't know	345	57.5

**How much force must be applied during chest compression?**		

Enough that the rib cage moves down 1 cm to 2 cm	203	33.8

Moderate force, such that the rib cage moves down 5 to 6 cm	267	44.5

High force, such that the rib cage moves down 6 cm to 10 cm	42	7.0

As much force as possible	88	14.7

**What do you know about the device defined as a defibrillator that is used during the chest compression when necessary?**		

I have never heard of it	75	12.5

I have heard of it before but not seen it	96	16

It is a device supporting respiration	55	9.2

It is a device used to restart a heart that has stopped working	428	71.3

**Do you have any idea about where an ‘automated external defibrillator' or a ‘pacemaker' can be found?**		

I don't know	468	78.0

Yes	132	22.0

**Table 8:** Association between identification of cardiac arrest findings and CPR training.

**Variable **	**Those who have not received CPR training** **N** %	**Those who have received CPR training** **N** %	**Total** **N** %	**p value** ^**a**^
**Consciousness evaluation**				

No response when called	126	42	168	0.151
	29.4%	24.4%	28.0%	
No response when touched	25	13	38	
	5.8%	7.6%	6.3%	
Not moving at all	49	14	63	
	11.4%	8.1%	10.5%	
Those knowing three of the procedures correctly	48	29	77	
	11.2%	16.9%	12.8%	
Those knowing two of the procedures correctly	102	49	151	
	23.9%	28.5%	41.4%	
Those who do not know any of the procedures (I don't know)	78	25	103	
	18.2%	14.5%	17.2%	

**Respiration evaluation**				

No respiratory movement	90	34	124	0.018*∗*
	21.0%	19.7%	20.7%	
No respiratory sound	35	7	42	
	8.2%	4.1%	7.0%	
No air coming out of the mouth of an individual	55	18	73	
	12.9%	10.5%	12.2%	
No steaming up of a mirror placed in front of the mouth of an individual	17	7	24	
	4.0%	4.1%	4.0%	
Those knowing three of the procedures correctly	66	49	115	
	15.4%	28.5%	20.2%	
Those knowing two of the procedures correctly	133	55	188	
	31%	32%	31.2%	
Those who do not know any of the procedures (I don't know)	29	9	38	
	6.8%	5.2%	6.3%	

**Circulation evaluation**				

Not feeling a pulse in the vessels of the neck	91	30	121	0.026*∗*
	21.3%	17.4%	20.2%	
Not feeling a pulse in the vessels of the arm	135	52	187	
	31.5%	30.2%	31.2%	
Both	120	73	193	
	28.0%	42.4%	32.2%	
I don't know	91	17	108	
	21.3%	9.9%	18%	

a: Respondents who chose ‘I do not know' were excluded from the statistical test of significance

**Table 9:** Association between the actions performed to a witnessed cardiac arrest with previous CPR training.

**Variable **	**Those who have not received CPR training** **N** %	**Those who have received CPR training** **N** %	**Total** **N** %	**p value**
I both gave chest compressions and conducted mouth-to-mouth ventilation (i.e., I gave CPR)	15	21	36	0.568
	16.3%	43.8%	22.1%	
I called emergency services	57	22	79	
	62.0%	45.8%	75.0%	
I just watched and left	20	5	25	
	21.7%	10.4%	17.9%	

**Table 10:** Association of CPR application with previous CRP training.

**Variable **	**Those who have not received CPR training** **N** %	**Those who have received CPR training** **N** %	**Total** **N** %	**p value**
**What BLS applications can you apply?**				

I can open the airway	39	19	58	0.0001*∗∗*
	9.1%	11.0%	9.7%	
I can control respiration	6	1	7	
	1.4%	0.6%	1.2%	
I can ventilate/conduct mouth-to-mouth ventilation	32	23	55	
	7.5%	13.3%	9.2%	
I can give chest compression	43	27	70	
	10.0%	15.7%	11.7%	
I can both ventilate and give chest compression	95	82	177	
	22.2%	47.7%	29.5%	
I do not know	215	18	233	
	50.2%	10.5%	38.8%	

**What is the proper rate of chest compression /artificial ventilation during the CPR (compressions/breaths)?**				

5/1	122	51	173	0.0001*∗∗*
	28.5%	29.6%	28.8%	
15/2	79	50	131	
	18.4%	29%	21.8%	
30/2	23	46	69	
	5.3%	26.7%	11.5%	

**Where must chest compression be applied?**				

An upper part of the chest	82	28	110	0.0001*∗∗*
	19.2%	16.3%	18.3%	
Middle of the chest	222	111	333	
	51.9%	64.5%	55.5%	
A lower part of the chest	52	31	83	
	12.1%	18.0%	13.8%	
Other	72	2	74	
	16.8%	1.2%	12.3%	

**What must be the rate of the chest compression?**				

At least 150 times per minute	12	10	22	0.0001*∗∗*
	2.8%	5.8%	3.7%	
At least 100 times per minute	32	39	71	
	7.5%	22.7%	11.8%	
At least 50 times per minute	90	72	162	
	21.0%	41.9%	27.0%	
I do not know	294	51	345	
	68.7%	29.7%	57.5%	

**How much force must be applied during chest compression?**				

Enough that the rib cage moves down 1 cm to 2 cm	133	68	203	**0.004** **∗**
	31.0%	39.5%	33.8%	
Moderate force, such that the rib cage moves down 5 cm to 6 cm	185	84	267	
	43.3%	48.8%	44.5%	
High force, such that the rib cage moves down 6 cm to 10 cm	35	7	42	
	8.2%	4.1%	7.0%	
As much force as possible	75	13	88	
	17.5%	7.6%	14.7%	

## Data Availability

The data used to support the findings of this study are available from the corresponding author upon request.

## Additional Points

*Limitations.* This study has a few limitations that are very important to consider in its interpretation and in the context of further research efforts. One of the major weak points in this research is that, during the filling out of the questionnaire under our supervision, many of the participants asked us what to do in the case of some of the questions that did not have the answer choice of ‘I have not seen this' or ‘I have no idea.' Furthermore, a few of the participants asked us for more details about the meaning of some sentences that were not clear enough to them. We tried to explain the meaning of some choices to them, so we expect that some of the responses may be biased as a result of this. Moreover, this study was conducted only in a very limited area (the city of Jeddah), so the results may vary in other Saudi cities or countries.

## Supplementary Materials

Supplementary Materials Supplementary material contains the questionnaire given to the participants. The questionnaire included 22 questions in Arabic and English language.
