# Supplementary material for: Knowledge of Nonmedical Individuals about Cardiopulmonary Resuscitation in Case of Cardiac Arrest: A Cross-Sectional Study in the Population of Jeddah, Saudi Arabia
Source: Emerg Med Int. 2019 Jan 16;2019:3686202. doi: 10.1155/2019/3686202 (PMC6354151; doi:10.1155/2019/3686202)
Supplement: Supplementary Materials — Supplementary material contains the questionnaire given to the participants. The questionnaire included 22 questions in Arabic and English language. [file 3686202.f1.pdf]

أ- **المعلومات السكانية:**

- **يرجى ذكر عمرك:**

- **يرجى ذكر الجنس:** ☐ ذكر ☐ انثى

- **يرجى ذكر مستوى تعليمك:** ☐ الابتدائي ☐ المتوسط ☐ الثانوي ☐ كلية

☐ جامعي ☐ متخرج من الجامعة

- **يرجى ذكر مهنتك:**

☐ موظف\ة ☐ عامل\ة ☐ طالب\ة ☐ ربة منزل

☐ متقاعد\ة ☐ عاطل عن العمل

1- **أي مما يلي قد يكون علامة من علامات السكتة القلبية المفاجئة؟ (يمكنك اختيار أكثر من خيار واحد)** :

☐ فقدان الوعي ☐ توقف التنفس ☐ توقف الدورة الدموية ☐ إزرقاق في الشفايف ☐ الغثيان

☐ صعوبة في التنفس ☐ آلام في الصدر ☐ شحوب في لون الجلد ☐ عدم وجود ايه حركة

2- **كيف يمكن معرفة حالة وعي الفرد؟ (يمكنك اختيار أكثر من خيار واحد)** :

☐ لا يستجيب عند النداء ☐ لا يستجيب عند اللمس ☐ لا يتحرك نهائياً ☐ لا اعرف

3- **كيف يمكن معرفة توقف عملية التنفس لدى الفرد ؟ (يمكنك اختيار أكثر من خيار واحد):**

☐ عدم وجود أي حركة في الجهاز التنفسي

☐ عدم وجود أي صوت تنفسي

☐ لا يوجد هواء خارج من فم الفرد

☐ لا يوجد غباشة لمرآة وضعت أمام فم الفرد

☐ لا اعرف

4- **كيف يمكن معرفة توقف الدورة الدموية؟ (يمكنك اختيار أكثر من خيار واحد):**

☐ عدم احساس بنبض في الأوعية التي في الرقبة

☐ عدم احساس بنبض في الأوعية التي في الذراع

☐ لا اعرف

5-هل سبق لك أن شاهدت الموت المفاجئ؟ إذا كان الجواب نعم، من كان / هي؟ (يمكنك اختيار أكثر من خيار واحد) :

☐ شخص من عائلتي

☐ شخص من أصدقائي أو معارفي

☐ شخص غريب

☐ لم ارى

6-إذا كنت قد شاهدت مثل هذا الحدث، ماذا فعلت في تلك الحالة؟ (إذا لم ترد على السؤال السابق، تخطي هذا السؤال) (يمكنك اختيار أكثر من خيار واحد):

- ☐ بدأت في التدليك القلبي
- ☐ بدأت في اعطاء التنفس الفموي
- ☐ بدأت في التدليك القلبي و في اعطاء التنفس الفموي (الانعاش القلبي الرئوي)
- ☐ اتصلت بسيارة إسعاف
- ☐ طلبت من شخص ما ان يطلب المساعدة
- ☐ لقد طلبت المساعدة عن طريق الهاتف
- ☐ أنا فقط شاهدت و غادرت

7-ما هو برأيك "التدليك القلبي" ؟

- ☐ فرك الصدر على فترات معينة
- ☐ تطبيق ضغط قوي على الصدر على فترات معينة (ضغط)
- ☐ فرك القلب مباشرة بعد فتح جدار الصدر
- ☐ تطبيق ضغط مباشرة على القلب بعد فتح جدار الصدر
- ☐ ليس لدي أي فكرة

8- إذا حدث الموت المفاجئ في احدى الأشخاص التاليين، لمن ستجري التنفس وتبدأ في التدليك القلبي؟(يمكنك اختيار أكثر من خيار واحد):

- ☐ شخص من العائلة
- ☐ صديقك
- ☐ جارك
- ☐ شاب في الصالة الرياضية
- ☐ غريب في السوبر ماركت
- ☐ شخص نظافته الشخصية قليلة ومتواجد في محطة للحافلات
- ☐ ولد يتعاطى المخدرات ويستخدم الغراء والحشيش والهيروين

9-إذا شخص ما من بين أفراد عائلتك او الأصدقاء اغمى عليه (الموت المفاجئ) ماذا ستفعل؟

- ☐ سأبدأ في إعطاء التدليك القلبي
- ☐ سوف استدعي سيارة إسعاف
- ☐ سوف أدعو شخص ما أو اطلب المساعدة
- ☐ أود فقط مشاهدة ومن ثم المغادرة

10- ماذا ستفعل إذا شاهدت شخصاً غريبا اغمى عليه (الموت المفاجئ) ؟

☐ سأبدأ في إعطاء التدليك القلبي

☐ سوف استدعي سيارة إسعاف

☐ سوف أدعو شخص ما أو اطلب المساعدة

☐ أود فقط مشاهدة ومن ثم المغادرة

11- ما هي المخاوف التي قد تمنعك من إعطاء التدليك القلبي للأصدقاء أو للأقارب؟

☐ إرتكب خطأ

☐ تسبب في كسور العظام

☐ تسبب في ضرر اللأعضاء

☐ توقف قلب عن العمل

☐ العقوبة لأسباب قانونية

☐ التلوث بالدم أو القيء

☐ العدوة بمرض معدي

☐ اسباب اخرى

12- ما هي المخاوف التي قد تمنعك من إعطاء التدليك القلبي للغريب؟

☐ إرتكب خطأ

☐ تسبب في كسور العظام

☐ تسبب في ضرر اللأعضاء

☐ توقف قلب عن العمل

☐ العقوبة لأسباب قانونية

☐ التلوث بالدم أو القيء

☐ العدوة بمرض معدي

☐ اسباب اخرى

13-هل تعرف كيفية إعطاء التدليك القلبي في حالة السكتة القلبية وتوقف الجهاز التنفسي (الموت المفاجئ)؟

☐ نعم   ☐ لا

14-- هل تلقيت أي تدريب في هذا الموضوع؟ (مبادئ الانعاش القلبي الرئوي).

☐ نعم   ☐ لا

15-إذا كان ردك هو نعم على السؤال السابق ، من أين تلقيت التدريب؟

- ☐ في المدرسة
- ☐ في الجامعة
- ☐ أثناء خدمتي العسكرية
- ☐ خلال مدرسة لتعليم القيادة
- ☐ في دورة مجتمع الإنعاش
- ☐ في دورة من قبل المدربين من وزارة الصحة
- ☐ في دورة تعطى من قبل البلدية
- ☐ في نادي رياضي
- ☐ في دورة معينة في مكان العمل
- ☐ تلفزيون- الانترنت - وسائل الإعلام

16- إذا كنت تواجه شخص توقف قلبه، أي من اساسيات الانعاش القلبي الرئوي يمكنك تطبيقه ؟

- ☐ يمكنني فتح مجرى الهواء
- ☐ يمكنني التحكم في التنفس
- ☐ يمكنني اعطاء التنفس الفموي
- ☐ أستطيع البدء في تدليك القلبي
- ☐ يمكنني اعطاء التنفس الفموي و البدء في تدليك القلبي
- ☐ لا اعرف

17- ما هو المعدل المناسب لتدليك القلبي/ التهوية الاصطناعية أثناء التدليك ؟

- ☐ **1\5**
- ☐ **2\15**
- ☐ **2\30**
- ☐ اخر

18-اي من الخيارات هو افضل مكان للقيام بالتدليك القلبي ؟

- ☐ الجزء العلوي من الصدر
- ☐ منتصف الصدر
- ☐ الجزء السفلي من الصدر

- [ ] آخر

19-كم يجب أن يكون معدل التدليك القلبي؟

- [ ] على الأقل 150 مرة في الدقيقة الواحدة
- [x] على الأقل 100 مرة في الدقيقة الواحدة
- [ ] على الأقل 50 مرة في الدقيقة الواحدة
- [ ] لا اعرف

20--كم القوة التي يجيب تطبيقها اثناء التدليك القلبي ؟

- [ ] يكفي أن القفص الصدري يتحرك الى الأسفل بمسافة 1 إلى 2 سم
- [x] قوة معتدلة، بحيث يتحرك القفص الصدري الى الأسفل بمسافة 5 إلى 6 سم
- [ ] قوة عالية، بحيث ان القفص الصدري يتحرك الى الأسفل بمسافة 6-10 سم
- [ ] أكبر قدر ممكن من القوة

21--ماذا تعرف عن الجهاز الذي يعرف بأنه جهاز صدمات الكهرائية الخارجي الذي يستخدم خلال التدليك القلبي عند الضرورة؟ (يمكنك اختيار أكثر من خيار واحد)

- [ ] لم اسمع به من قبل
- [ ] لقد سمعت من قبل ولكن لم أراه
- [ ] هو جهاز لدعم التنفس
- [x] هو جهاز لإعادة تشغيل قلب الذي توقف عن العمل

22-هل لديك أي فكرة عن مكان " جهاز صدمات الكهرائية الخارجي " أو "جهاز تنظيم ضربات القلب" يمكن العثور عليها؟

- [ ] نعم
- [ ] لا أعرف

A-Demographic information:

Please state your age:

Please state you gender: ☐ Male ☐ Female

Please state your education: ☐ Elementary ☐ intermediate ☐ High school ☐ College ☐ University ☐ Post-graduate

Please sate your occupation:

☐ Employee ☐ Worker ☐ Student ☐ House wife ☐ Retired ☐ Unemployed

1-Which of the following may be sign of sudden cardiac arrest? (You can choose more than one option)

☐ Loss of consciousness ☐ Discontinuation of breathing ☐ Discontinuation of circulation ☐ Cyanosis ☐ Nausea ☐ Difficulty in breathing ☐ Chest pain ☐ Faintness of the skin ☐ The individual is not moving.

2-how can the consciousness state of the individual be determined? (You can choose more than one option)

☐ No response when called ☐ No response when touched ☐ Not moving at all ☐ I don't not know

3-how can the absence of respiratory be determined? (You can choose more than one option)

☐ Not having any respiratory movement

☐ Not having any respiratory sound

☐ Not coming air out of the mouth of individual

☐ Not steaming up a mirror placed in front of the mouth of individual

☐ I do not know

4-how can the absence of circulation be determined? (You can choose more than one option)

☐ Not feeling a pulse in the vessels of the neck

☐ Not feeling a pulse in the vessels of the arm

☐ I don't know

5-Have you ever witnessed a sudden death? If yes, who was he/she? (You can choose more than one option)

☐ Somebody from my family

☐ Somebody from my friends or acquaintances

☐ A stranger

☐ I have not seen this

6-if you have witnessed such an event, what did you do in the situation? (If you replied no to the previous question, skip this question) (You can choose more than one option).

- ☐ I began to give chest compressions
- ☐ I conducted mouth to mouth ventilation
- ☐ I both gave chest compressions and conducted mouth to mouth ventilation (I gave CPR)
- ☐ I called an ambulance
- ☐ I told somebody to call for help
- ☐ I called for help by telephone
- ☐ I just watched and left

7-what do you think a "chest compression" means?

- ☐ To scrub the chest at certain intervals
- ☐ To apply strong compression to the chest at certain intervals (compress)
- ☐ To scrub the heart directly opening the chest wall
- ☐ To apply compression directly to the heart opening the chest wall
- ☐ I have no idea

8- If sudden death occurs in the following people, for whom would you conduct respiration and give chest compression?

- ☐ Someone from the family
- ☐ Your friend
- ☐ Your neighbor
- ☐ A youth in the sports hall
- ☐ A stranger in the supermarket
- ☐ A person who has poor personal hygiene at the bus stop
- ☐ A gamin who is drug dependent and uses glue, hashish and heroin

9-if somebody from among your family members or friends felt faint (sudden death) what would you do?

- ☐ I would begin to give chest compression
- ☐ I would call an ambulance
- ☐ I would call somebody or call for help
- ☐ I would just watch and leave

10-what would you do if you witness a stranger feeling faint (sudden death)

☐ I would begin to give chest compression

☐ I would call an ambulance

☐ I would call somebody or call for help

☐ I would just watch and leave

11-what concerns may prevent you from giving chest compression to you friends or relatives?

☐ Making a mistake

☐ Causing bone factures

☐ Causing harm to organs

☐ Stopping a working heart

☐ Punishment due to legal reasons

☐ Contamination by blood or vomiting

☐ Contracting a contagious disease

☐ Other

12- What concerns may prevent you from giving chest compression to stranger?

☐ Making a mistake

☐ Causing bone factures

☐ Causing harm to organs

☐ Stopping a working heart

☐ Punishment due to legal reasons

☐ Contamination by blood or vomiting

☐ Contracting a contagious disease

☐ Other

13-do you know how to give chest compression in case of cardiac arrest and respiratory standstill (sudden death)?

☐ Yes ☐ No

14- Have you received any training in this subject? (Basic life support training).

☐ Yes ☐ No

15) If your replay is yes to the above question, where did you receive the training?

☐ At school

☐ At university

- [ ] During my military service
- [ ] During the driving school
- [ ] At a resuscitation society course
- [ ] At a course given by the trainers of the ministry of health
- [ ] At a course given by the municipality
- [ ] In a sports club
- [ ] At a course given in the workplace
- [ ] Television –internet- media
- [ ] Other

16-if you are confronted with a person whose heart has stopped, which of the basic life support applications can you apply?

- [ ] I can open the airway
- [ ] I can control respiration
- [ ] I can ventilate/conduct mouth to mouth ventilation
- [ ] I can give chest compression
- [ ] I can both ventilate and give chest compression
- [ ] I don't know

17-what is the proper rate of chest compression /artificial ventilation during massage?

- [ ] 5/1
- [ ] 15/2
- [x] 30/2
- [ ] Other

18-which are must chest compression be applied on?

- [ ] Upper part of the chest
- [x] Middle of the chest
- [ ] Lower part of the chest
- [ ] Other

19-what must be the rate of the chest compression?

- [ ] At least 150 times per minute
- [x] At least 100 times per minute
- [ ] At least 50 time per minute

☐ I don't know

20-how much force must be applied during heart massage?

☐ Enough that the rib cage moves down 1 to 2 cm

☐ Moderate force, such that the rib cage moves down 5 to 6 cm

☐ High force, such that the rib cage moves down 6 to 10 cm

☐ As much force as possible

21-what do you know about the device defined as defibrillator that us used during chest compression when necessary? (You can choose more than one option)

☐ I have never heard of it

☐ I have heard of it before but not seen it

☐ It is a device supporting respiration

☐ It is device to restart a heart the has stopped working

22-do you have any idea about where an "automated external defibrillator " or "pace maker" can be found?

☐ Yes ☐ I don't know
